# In Vivo Study on 3D-Printed Polylactic Acid Nerve Tubes for Sciatic Nerve Injury Treatment

**DOI:** 10.3390/polym17141992

**Published:** 2025-07-21

**Authors:** Salih Kavuncu, Rauf Hamid, Ömer Faruk Sarıahmetoğlu

**Affiliations:** 1 Department of Plastic Reconstructive and Aesthetic Surgery, Medical Faculty, Afyonkarahisar Health Sciences University, 03030 Afyonkarahisar, Türkiye; salih.kavuncu@afsu.edu.tr; 2 Department of Radiology, Cerrahpasa Medical Faculty, Istanbul University-Cerrahpasa, 34320 Istanbul, Türkiye; omer.sariahmetoglu@iuc.edu.tr

**Keywords:** 3D printing, nerve degeneration, nerve conduit, nerve repair

## Abstract

**Background/Objectives:** Nerve injuries cause functional loss and psychosocial issues due to prolonged rehabilitation. Recently, 3D-modeled nerve conduits have been used to aid in surgical planning. This study investigated the impact of 3D-bioprinted PLA, chitosan, alginate, and collagen conduits on nerve regeneration in a rat sciatic nerve crush injury model. **Methods**: This study, conducted at Kütahya University of Health Sciences, involves 50 rats were divided into four groups: (1) sham-operated controls, (2) sciatic nerve injury without treatment, (3) injury treated with a PLA conduit, and (4) injury treated with 3D-printed tubes composed of chitosan and alginate. The procedures were performed, blood was collected, and the rats were sacrificed after two months. Weekly checks for infection, scar healing, and motor responses were performed. **Results**: Rats with nerve conduits showed less macroscopic scarring. Weekly assessments of motor nerve recovery showed no movement restrictions in limbs treated with PLA conduits, graft conduits, or conduits bridging retracted nerve stumps, based on responses to stimulus checks. An infection developed in the sciatic nerve and surrounding muscle tissue of one rat with a bio-graft conduit, prompting histopathological examination to investigate its cause. **Conclusions**: This proof-of-principle study demonstrates the feasibility of using 3D-printed biocompatible nerve conduits for peripheral nerve repair, providing a basis for future, more comprehensive investigations.

## 1. Introduction

Peripheral nerve injuries are frequently encountered in clinical practice, with an annual incidence of 1/1000 worldwide [1]. After injury from stretching, crushing, or cutting, significant histopathological changes occur, leading to axonal damage and impaired impulse transmission within 48–96 h [2]. This results in neurological dysfunction due to failed signal transmission to muscles or sensory organs [3]. Blood flow disruption due to nerve compression, followed by reperfusion, generates acute-phase reactants (such as TNF-alpha and IL-1 beta) and free oxygen radicals.

Despite advancements in neurotrophic drugs, steroids, hormones, low-dose radiation, and microsurgical techniques, full motor and sensory recovery in nerve regeneration remains elusive [1,2,3,4,5]. The peripheral nervous system is more successful in axonal regeneration owing to fewer inhibitory myelin proteins and more active Schwann cells distally. Agents believed to enhance nerve regeneration are commonly used in experimental peripheral nerve injury studies [6,7]. Although effective, the side effects of methylprednisolone and gabapentin have been debated [5].

Erythropoietin (EPO), which is found in the liver and kidneys, exerts neuroprotective effects following nerve injury. Although beneficial in peripheral neuropathy models [6,7], no experimental studies in various peripheral nerve injury models (crush and full-cut) have been reported.

Despite being the gold standard, autologous nerve grafts have several disadvantages. Tube-like materials can fill this gap in the treatment of peripheral nerve injury. However, synthetic nerve tubes carry a risk of infection and are not biocompatible. Advancing 3D bioprinting technology offers fully biological, durable, and infection-risk-reducing structures tailored via computer modeling [8]. Ongoing in vivo and in vitro studies have explored nerve regeneration and bone repair using gel-phase biofilaments made from various chemical materials (fibrin, collagen, polycaprolactone, alginate, laminin, and gelatin).

Three-dimensional bioprinting is a rapidly evolving technique in tissue engineering, enabling the fabrication of complex, cell-laden scaffolds through layer-by-layer deposition. The primary bioprinting modalities include extrusion-based, jetting-based, and vat photopolymerization-based techniques, each offering unique advantages and limitations depending on the material properties and biological goals. Extrusion bioprinting, the most widely adopted method, accommodates high-viscosity bioinks and allows for cell-dense, macroscale constructs, but generally suffers from lower resolution and higher shear stress on cells during extrusion [9]. Jetting-based bioprinting employs drop-on-demand strategies to precisely deposit low-viscosity droplets with minimal shear stress, making it suitable for delicate cell types and high-resolution patterning, albeit with viscosity and print volume constraints [10]. Vat photopolymerization techniques, including stereolithography (SLA) and digital light processing (DLP), achieve ultra-high resolution by selectively curing photoactive bioresins using light, enabling the creation of intricate tissue architectures; however, material compatibility and cytocompatibility of photoinitiators remain key concerns [11]. Understanding the principles and trade-offs of these bioprinting techniques is essential for tailoring constructs to specific regenerative applications.

In the evolving field of peripheral nerve regeneration, there is a clear shift from the use of pristine polymeric scaffolds toward multifunctional polymer composite systems. These materials are engineered to address the complex biological requirements of nerve repair by combining structural support with therapeutic, conductive, or regenerative functionalities. Composite systems may include drug-loaded polymers for the localized delivery of anti-inflammatory or neurotrophic agents, polymer–protein conjugates that mimic the extracellular matrix, ionically or electrically conductive polymers to enhance axonal signaling, stem-cell-laden scaffolds for cellular replacement, or polymer–bioglass hybrids that promote osteoneural integration in specific anatomical contexts. Additionally, nanofiber-reinforced polymers, growth factor-immobilized matrices, and immunomodulatory polymer blends have emerged as promising tools in next-generation nerve repair strategies. The increasing diversity and functionality of these composite approaches aim to overcome the limitations of single-material systems and align more closely with the multifactorial demands of tissue engineering in neuroregeneration [12,13,14,15].

PLA, chitosan, alginate, and collagen were selected due to their favorable physicochemical properties and previous evidence supporting their use in peripheral nerve regeneration. PLA is a biocompatible, biodegradable thermoplastic that offers sufficient mechanical strength and has been approved for various biomedical applications by regulatory bodies such as the FDA [16,17,18]. Chitosan, a natural polysaccharide derived from chitin, promotes Schwann cell adhesion and axonal regeneration, while also exhibiting antibacterial properties [19,20,21]. Alginate, a naturally occurring anionic polymer, forms hydrogels in the presence of divalent cations and supports nutrient diffusion and cellular growth [22,23,24]. Collagen, a major component of the extracellular matrix, facilitates cell migration and neurite extension and has been widely used in nerve guidance conduits due to its excellent biocompatibility and biodegradability [25,26]. The combination of these materials aims to create a structurally stable yet bioactive environment conducive to axonal repair. However, other biocompatible polymers such as polycaprolactone (PCL), polyglycolic acid (PGA), polylactic-co-glycolic acid (PLGA), and poly(3-hydroxybutyrate-co-3-hydroxyvalerate) (PHBV) have also been investigated for peripheral nerve conduit fabrication [27]. In addition, composite polymers combining PLA with other bioactive fillers or nanomaterials, such as carbon nanotubes, graphene, or hydroxyapatite, have also been explored in nerve conduit fabrication to improve mechanical strength and biological interactions [28]. However, their clinical translation is still limited, and PLA alone remains a promising candidate due to its ease of processing and regulatory status. PLA was specifically chosen for this proof-of-principle study based on its ease of 3D printing, favorable degradation profile, and regulatory acceptance. Additionally, while various additive manufacturing techniques, including electrospinning, stereolithography, and digital light processing, have been explored to develop nerve guidance conduits with tailored properties [9,29], there is still a lack of in vivo evidence demonstrating the feasibility of using FDM-printed PLA-based conduits in a sciatic nerve crush model, which this study aimed to address. The combination of these biomaterials in a 3D-printed design was hypothesized to create a structurally stable yet bioactive environment conducive to axonal repair, potentially offering a customizable and biocompatible alternative for peripheral nerve regeneration.

## 2. Materials and Methods

All animals were housed under standard laboratory conditions with a 12 h light–dark cycle and free access to food and water. Ethical approval for this study was obtained from the Kütahya University of Health Sciences Animal Care and Use Committee (approval number 2019.01.05).

### 2.1. Bioink Preparation Protocol

The extracellular scaffold matrix (ESM)-based bioink used in this study was prepared by combining collagen, alginate, and chitosan. Each component was prepared under sterile conditions and mixed to achieve optimal viscosity for bioprinting. Specifically, the bioink consisted of type I collagen (rat tail, ~3 mg/mL; cat. no. 354249, Corning Inc., Corning, NY, USA) to provide a biocompatible structural base, sodium alginate (2% *w/v*; cat. no. A2158, Sigma-Aldrich, St. Louis, MO, USA) as a gel-forming agent contributing to the hydrogel scaffold, and chitosan (1% *w/v*, low molecular weight; cat. no. 448869, Sigma-Aldrich, St. Louis, MO, USA) for its antibacterial properties and support for Schwann cell adhesion.

The materials were gently mixed at room temperature using a magnetic stirrer for 30 min to achieve a homogenous solution. The resulting bioink was loaded into a sterile 3 mL syringe and used immediately for bioprinting under UV-C sterilized conditions to prevent contamination. The mixture was optimized for extrusion at a working pressure of 7 psi and a layer thickness of 0.1 mm during printing.

### 2.2. Bioprinting Procedure

In our study, to produce biotubes for use in sciatic nerve injuries, we utilized Ultimaker 2 and Ultimaker 3 Extended 3D printers (Ultimaker B.V., Utrecht, The Netherlands) and the Ultimaker Cura software (Cura, version 3.3.1; Ultimaker B.V., Utrecht, The Netherlands) available at Kütahya University of Health Sciences Application and Research Center (KUYAM).

**Figure 1:**
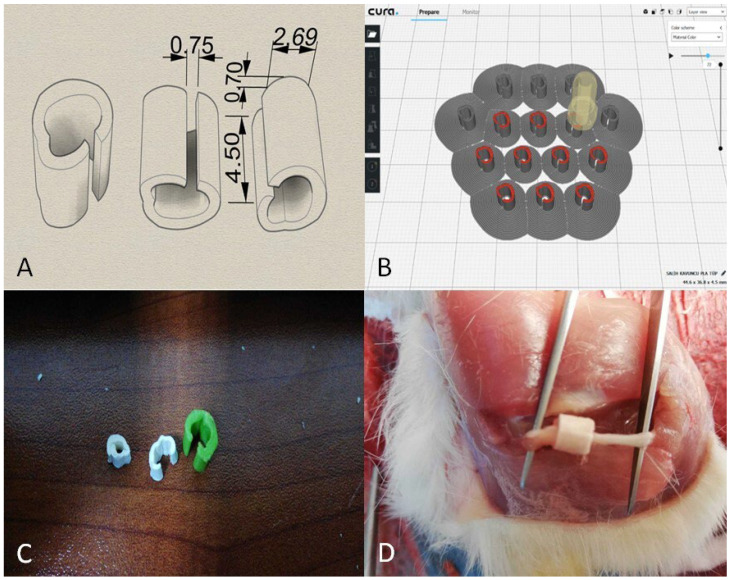
Preparation of printing settings. (**A**) Dimensional schematic of the PLA nerve conduit. Internal diameter = 2.69 mm; length = 4.5 mm; notch width = 0.75 mm; tab thickness = 0.7 mm. (**B**) Slicing preview in Ultimaker Cura showing layer height = 0.2 mm. (**C**) Photograph of the printed PLA conduits in various sizes. (**D**) Intraoperative image showing the placement of the PLA conduit on the transected sciatic nerve.

The PLA tubes prepared for this study were designed by considering factors such as the surgical operation conditions and maintenance of their position post-operatively. The incisions made during the operation were sized to fit the smallest possible implants, ensuring that the PLA tubes covered the entire nerve damage area, extending at least 1 mm beyond the edges of the damaged area for proper integration. Initially conceived as cylindrical structures, PLA tubes were modified accordingly. To attach the tubes directly to the damaged area without further intervention during surgery, a vertical cut and small notch were designed to provide graduated compression once fitted. This modification allowed the surgeon to easily secure the tubes around the nerve without causing additional damage or adjusting tightness for proper fixation. To produce biotubes, we used an Ultimaker 3 Extended 3D printer (Ultimaker B.V., Utrecht, The Netherlands) and Ultimaker Cura v. 3.3.1 software (Ultimaker B.V., Utrecht, The Netherlands). PLA tubes were designed in SolidWorks 2020 to cover the nerve crush site plus 1 mm margins on either side (internal diameter = 2.69 mm; length = 4.5 mm; notch width = 0.75 mm; tab thickness = 0.7 mm). Each design was saved as an STL file, smoothed in Autodesk Meshmixer (Autodesk Inc., San Rafael, CA, USA), and imported into Cura (Figure 1).

PLA (d = 1.25 g/cm^3^) was used as a filament for printing. The printing parameters of the PLA filaments are presented in Table 1. After these steps, the spine and tumor structures to be printed were obtained in a G-Code format and transferred to the printer via Wi-Fi or USB. The spine printing process took approximately two days.

The neural conduits were fabricated using a fused deposition modeling (FDM) 3D printer (Ultimaker 3 Extended, Ultimaker B.V., Utrecht, The Netherlands) and a commercially available PLA filament (Ingeo™ 4032D; NatureWorks LLC, Minnetonka, MN, USA). The filament had a molecular weight of approximately 200,000 g/mol, a diameter of 1.75 mm, and a density of 1.24–1.25 g/cm^3^. Printing was performed with a nozzle diameter of 0.4 mm at a nozzle temperature of 200 °C and a print bed temperature of 70 °C. The printing speed was set to 50 mm/s, and layer height was configured at 0.2 mm. A grid infill pattern was selected to ensure uniform internal support and mechanical integrity of the conduit wall. The infill density was set to 70%, balancing structural strength with material usage. The design was generated using SolidWorks, exported as an STL file, and prepared for slicing using Ultimaker Cura software. The printed conduits featured an open-slit design with a stabilizing notch for easy placement around the injured nerve. This geometry ensured proper coverage while maintaining mechanical stability during and after implantation (Figure 1A–C). During modeling, a model with a surface area of 2 cm^2^ and height of 3 mm was created, and ESM was chosen as the printing material (Table 1).

The PLA nerve conduits were fabricated using an Ultimaker-brand fused deposition modeling (FDM) 3D printer, as dedicated bioprinting systems capable of thermoplastic processing were unavailable during the experimental period in Türkiye. Although the Ultimaker system offered limited printing precision, numerous trial prints were performed to achieve the desired structural quality and dimensional stability. The following hydrogel solutions were prepared separately before application: chitosan solution (1% *w/v*) was dissolved in 0.1 M acetic acid to ensure solubility, collagen solution (1% *w/v*) was neutralized to pH 7.2–7.4, and alginate solution (1% *w/v*) was prepared in distilled water, then crosslinked with calcium chloride after mixing. These components were blended at a 33:33:33 ratio and homogenized under sterile conditions. Distilled water produced by a laboratory distillation unit was used for all preparations. The final hydrogel was extruded into the inner lumen of the PLA conduits to create a bioactive interface for nerve regeneration.

A hydrogel composite composed of chitosan, alginate, and type I collagen was prepared in a 1:1:1 ratio (*v*/*v*/*v*), consistent with literature-reported formulations for nerve regeneration scaffolds [31]. Specifically, chitosan solution (1% *w/v*) was dissolved in 0.1 M acetic acid to improve solubility, alginate solution (1% *w/v*) was prepared in distilled water and subsequently crosslinked with calcium chloride, and collagen solution (1% *w/v*) was neutralized to a pH of 7.2–7.4 prior to mixing. These solutions were blended under sterile conditions for 30 min to achieve homogeneity. The final hydrogel mixture was applied manually to the inner surface of the 3D-printed PLA conduits with an approximate coating thickness of 2–3 mm to provide a bioactive and hydrated environment favorable for axonal regeneration.

After checking the connections between the air compressor and bioprinter, the pressure inside the device was reduced to zero. The Petri dish for printing was placed in a slot on the plate. In the Repetier program, X-, Y-, and Z-axis calibrations of the device were performed considering the position of the Petri dish, and the origin point was determined. A 3 cc syringe containing ESM was placed in Extruder 1, and pneumatic connections were made. To ensure a sterile printing process, the control panel was closed and the cabinet was sterilized with UV-C radiation for 30 min. When printing began, the air pressure was gradually increased, and it was observed that 7 psi was the most suitable value for the material and model used. During printing, the layer thickness was set to 0.1 mm, and the printing speed was set to ‘low.’ The printing process was performed for 25 min.

### 2.3. Animal Experimentation

All animals used in this study were female Sprague–Dawley rats (250–300 g, 8–10 weeks old) obtained from Kütahya University of Health Sciences Application and Research Center (Kütahya, Türkiye). Female rats were selected to reduce aggressive behavior and minimize fighting-related injuries during post-operative care. Before the surgical procedure, general anesthesia was induced by intraperitoneal administration of 50 mg/kg ketamine hydrochloride (Ketasol 100 mg/mL, Richter Pharma AG, Wels, Austria) and 15 mg/kg xylazine (Rompun 2%, Bayer AG, Leverkusen, Germany). All surgical procedures were performed by the same surgeon, using a loop with 3.5× magnification.

Following intraperitoneal general anesthesia, the rats were placed in a prone position on the operating table to ensure surgical stabilization. The gluteal and thigh areas of the rats were shaved, and surgical field antisepsis was performed using povidone–iodine (Betadine, Purdue Products L.P., Stamford, CT, USA). The surgical area was covered with a sterile drape. After an oblique incision was made in the posterior left thigh, blunt dissection was performed, and the biceps femoris muscle was dissected to reach the sciatic nerve. Using loops and microsurgical instruments, the nerves were carefully dissected from the surrounding tissues without causing any damage.

In our study, following sciatic nerve dissection, an aneurysm clip (applying a constant pressure of 54 newtons) was kept on the sciatic nerve for 90 s to create a sciatic nerve crush injury.

**Figure 2:**
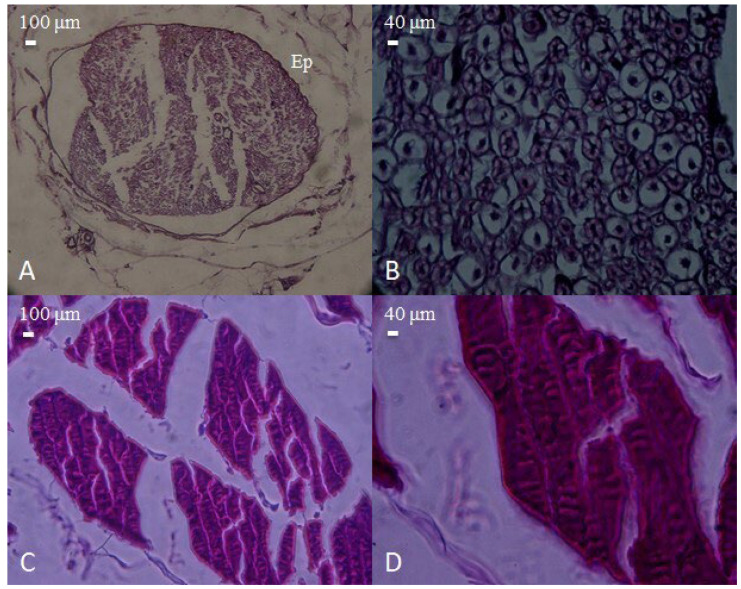
Microscopic appearance of peripheral nerve and skeletal muscle tissues obtained from the pre-injury control group. (**A**) Cross-section of a normal peripheral nerve encased in fibrous connective tissue (epineurium, Ep), showing intact fascicular architecture (H&E, 40×, scale bar = 100 μm). (**B**) Higher magnification of the nerve, demonstrating preserved axonal integrity and normal cellular morphology (H&E, 100×, scale bar = 40 μm). (**C**) Histological view of normal skeletal muscle bundles, exhibiting regular alignment and fiber integrity (H&E, 40×, scale bar = 100 μm). (**D**) Close-up of muscle fibers displaying typical striations and uniform organization (H&E, 100×, scale bar = 40 μm).

**Figure 3:**
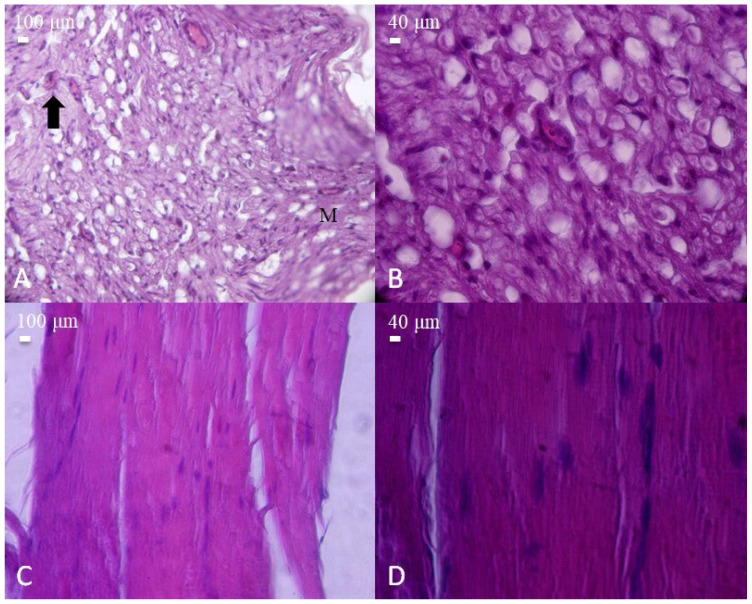
Histological appearance of nerve and muscle tissues from the post-injury control group (sciatic nerve transection without repair). (**A**) Cross-section of injured nerve showing prominent vacuolization and inflammatory cell infiltration (arrow). Macrophages (M) are observed among the nerve fibers (H&E, 40×, scale bar = 100 μm). (**B**) Higher magnification revealing severe axonal degeneration and cellular disruption (H&E, 100×, scale bar = 40 μm). (**C**) Longitudinal section of injured skeletal muscle fibers with disorganized structure and loss of alignment (H&E, 40×, scale bar = 100 μm). (**D**) Higher magnification displaying myofiber degeneration and nuclear displacement (H&E, 100×, scale bar = 40 μm).

**Figure 4:**
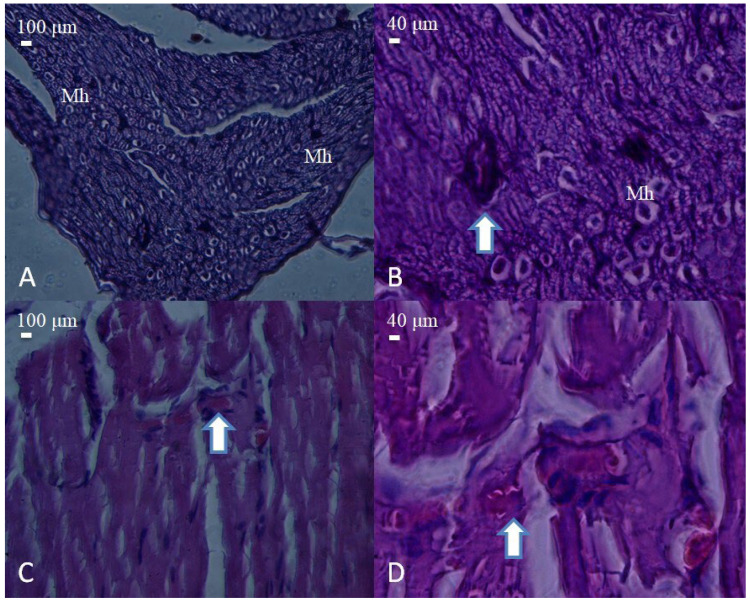
Histological appearance of nerve and muscle tissues from the PLA conduit group. (**A**) Cross-section of a nerve showing moderate structural organization and dense fascicular arrangement (H&E, 40×, scale bar = 100 μm). Mast cells (Mh) are observed among the nerve fibers. (**B**) Higher magnification reveals mild axonal disorganization and degenerative changes (arrow) (H&E, 100×, scale bar = 40 μm). (**C**) Muscle tissue with partially regenerated fibers and centrally located nuclei (arrow) (H&E, 40×, scale bar = 100 μm). (**D**) Detailed view showing degenerating myofibers with inflammatory cell infiltration (arrow) (H&E, 100×, scale bar = 40 μm).

**Figure 5:**
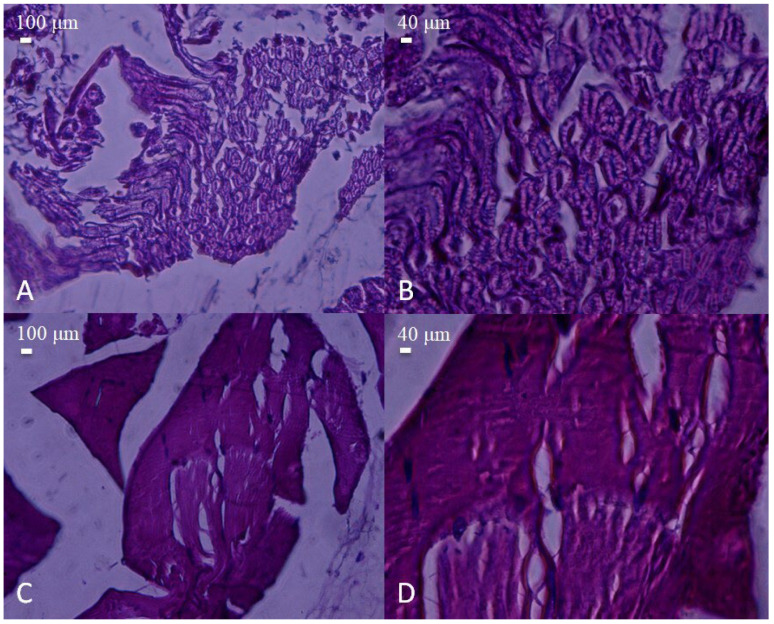
Histological appearance of nerve and muscle tissues from the biotube conduit group. (**A**,**B**) Nerve sections showing well-aligned myelinated fibers with relatively preserved architecture (H&E, A: 40×, scale bar = 100 μm; B: 100×, scale bar = 40 μm). (**C**,**D**) Muscle tissue displaying organized fiber bundles with mild structural irregularities (H&E, C: 40×, scale bar = 100 μm; **D**: 100×, scale bar = 40 μm). No significant inflammatory infiltration was observed compared to the PLA and injury groups.

The control group was classified as group 1 (Figure 2). After creating sciatic nerve injury in the rats, the wound was closed without implantation (Figure 3). After the procedure, blood samples were collected from the rats, and they were kept alive for two months. Rats with a sciatic nerve injury and PLA nerve tube implantation were designated as group 2 (Figure 4). After surgical implantation, blood samples were collected from the rats, and they were kept alive for two months. Rats with sciatic nerve injury and ESM bio nerve tube implantation were classified as group 3 (Figure 5). After surgical implantation, blood samples were collected from the rats, and they were kept alive for two months. At 8 weeks, the rats were euthanized under deep anesthesia (sodium pentobarbital, 150 mg/kg, i.p.; Nembutal, LIGAND Pharmaceuticals GmbH, Munich, Germany). Before sacrifice, the nerve and muscle tissues and blood samples were collected. Blood and muscle tissue samples were stored in formaldehyde (4%, neutral buffered, Merck, Darmstadt, Germany) at +4 °C until the biological and histological examinations were conducted. All blood samples were centrifuged at 4700 rpm for 10 min, and their sera were separated using a micropipette. Serum tubes were stored in formaldehyde at +4 °C until biological and histological examinations were conducted.

In similar preclinical studies investigating sciatic nerve regeneration, sample sizes of eight to twelve rats per group have been commonly reported [8,32]. This study included 40 rats (10 per group) to ensure scientific significance of the results and their statistical comparability. Preliminary statistics indicate that the number of rats was minimal. This study aimed to obtain meaningful results using a minimum number of rats.

### 2.4. Biological and Histological Studies

TNF-α and S100B ELISA (Double-Antibody Sandwich Enzyme-Linked Immunosorbent Assay) Analysis.

**Figure 6:**
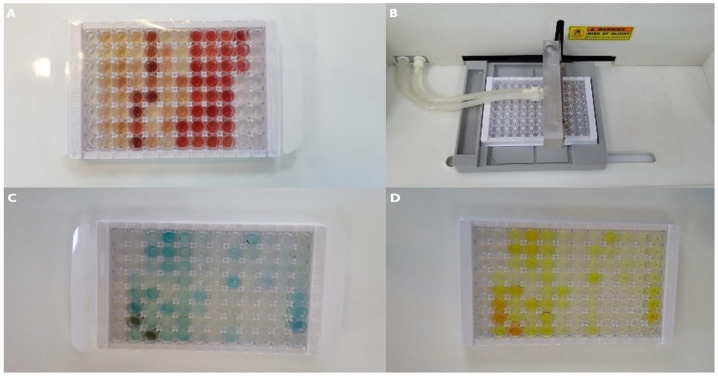
Representative images from ELISA procedures. (**A**) Standard serum samples loaded into a 96-well plate. (**B**) Automated plate washer used during washing steps. (**C**) Wells after substrate addition. (**D**) Wells after addition of stop solution to terminate the reaction.

To determine the TNF-α and S100B values for all groups, 5 mL blood samples were collected into tubes containing EDTA (BD Vacutainer, Franklin Lakes, NJ, USA). All blood serum samples were separated by centrifugation at 1000× *g* for 15 min. Serum samples were stored at −20 °C until ELISA experiments were conducted. The SEA133Ra commercial kit was used to determine TNF-α and S100B levels. Standard dilutions were prepared as follows: 100 μL from each of the standard dilution series and each sample was added to the wells, covered with a plate sealer, and incubated at 37 °C for 1 h (Figure 6A). After the incubation, the liquid in each well was removed. Detection Reagent A working solution (100 μL) was added to each well, covered with a plate sealer, and incubated at 37 °C for 1 h (Figure 6B). After incubation, the liquid in each well was removed and washed with 350 μL wash buffer. Detection Reagent B working solution (100 μL) was added to each well, covered with a plate sealer, and incubated at 37 °C for 30 min. After incubation, the wells were washed five times. Substrate solution (90 μL) was added to each well, each well was covered with a plate sealer, and they were incubated at 37 °C for 10–20 min (Figure 6C). Finally, 50 μL of the stop solution was added to each well and gently mixed (Figure 6D). The absorbance of the samples was measured at 450 nm wavelength using a ChoraMate microplate reader.

### 2.5. Statistical Analysis

For data evaluation, the SPSS 25 statistical package program (IBM Corp. Released 2017. IBM SPSS Statistics for Windows, Version 25.0. Armonk, NY, USA) was used. Variables are presented as the mean ± standard deviation. The normality of the variables and homogeneity of variances were checked (Shapiro–Wilk and Levene tests) before evaluation. When conducting data analysis, One-Way Analysis of Variance was used for comparisons of three or more groups, and a Tukey HSD test was used for multiple comparisons. If these conditions were not met, Kruskal–Wallis and Bonferroni–Dunn tests were used for multiple comparisons. The relationship between two continuous variables was evaluated using Pearson’s correlation coefficient; if the parametric test conditions were not met, Spearman’s correlation coefficient was used. The significance level for the tests was accepted as *p* < 0.05 and *p* < 0.01.

## 3. Results

### 3.1. TNF-α and S100B Levels in PLA and Biotube-Applied Serum Samples and Control Serum Samples for Nerve Recovery

When comparing the serum TNF-α values of rats with sciatic nerve injury treated with different methods to the control serum values, it was found that all groups, except for the serum group from blood samples taken after biotube implantation, had significantly higher values (*p* = 0.001). When comparing the serum S100B levels of rats with sciatic nerve injury treated with different methods to control serum values, it was determined that the serum samples taken after nerve injury, the serum samples collected two months after nerve injury, the serum samples taken after treatment with PLA tubes, and the serum samples collected after implantation were all significantly lower (*p* = 0.010). When comparing the serum S100B levels of the control group with those of the serum samples obtained after PLA tube implantation, it was found that the serum S100B levels were significantly higher after PLA tube implantation (*p* = 0.010). No difference was observed in the S100B values of serum samples taken after tube treatment compared with the control (Table 2).

### 3.2. Comparison of Serum TNF-α and S100B Values in PLA and Biotube Treatments After Nerve Injury

A positive correlation was found between TNF-α and S100B levels (rs = 0.777, *p* = 0.023) in serum samples collected after PLA tube treatment following nerve injury (Table 3).

No statistically significant correlation was found within the group between TNF-α and S100B levels in serum samples taken after nerve injury and those taken 2 months later, between serum TNF-α and S100B levels in blood samples taken after PLA tube implantation and 2 months of treatment, or between serum TNF-α and S100B levels in blood samples taken after biotube implantation and 2 months of treatment (Table 4).

## 4. Discussion

Peripheral nerve injuries often lead to prolonged healing and incomplete recovery, with functional impairment as the key outcome. Microsurgical techniques have improved repair; however, achieving full recovery remains a challenge. Initially, the nerve gaps were filled with donor sensory nerves, such as the sural nerve. Over time, synthetic materials have led to nerve guidance conduits, with the aim of creating the best environment for nerve regeneration. Autograft nerve tissue remains the gold standard; however, nerve conduits up to 3 cm are effective. In axonal injury, Wallerian degeneration occurs in the distal segment, and Schwann cells and macrophages clear debris, allowing for neurite extension. Correct alignment is crucial for preventing neuroma formation and disorganized nerve networks. Conduits, whether autografts or synthetic, are necessary to connect the proximal and distal ends.

The advent of 3D (three-dimensional) printing has enabled the creation of designs that are faster and less expensive than traditional methods. The addition of bioprinting to 3D models has advanced nerve regeneration studies by allowing for the inclusion of living cells, neurotrophic substances, and other biomaterials. We hypothesized that biotubes printed by bioprinting can enhance nerve regeneration.

In this study, a clip-shaped nerve conduit was designed to envelop injured yet anatomically continuous sciatic nerve fascicles without exerting pressure. Conventional manufacturing methods were unsuitable for such fine, anatomically scaled geometries, prompting the use of fused deposition modeling (FDM). Although the clip design challenged the limits of the Ultimaker 3 Extended printer, the iterative optimization of structural features—such as notch depth and hinge flexibility—enabled successful fabrication. PLA was chosen due to its low melting point, favorable biodegradation profile, and biocompatibility. These properties allowed the conduit to maintain mechanical integrity throughout the implantation period without compressing surrounding tissue. Upon degradation, PLA yields non-toxic lactic acid, which supports cellular proliferation and tissue healing [30,33].

Additionally, the conduit served as a protective barrier against external mechanical stress, while the flexibility of 3D printing enabled the rapid and cost-effective customization of the implant’s geometry to suit the animal model [9,29].

Although direct surface roughness measurements (e.g., via profilometry or atomic force microscopy) were not conducted, the surface characteristics of the 3D-printed PLA conduits were theoretically estimated based on established models [34,35]. According to the inclined surface model (Ra = h/2) and the empirical model (Ra = 0.045·h·(1 + w/h)), the average roughness (Ra) values were estimated at approximately 100 µm and 27 µm, respectively, given the printing parameters of 0.2 mm layer height and 0.4 mm nozzle diameter. The corresponding peak-to-valley height differences (Rz) were calculated to be ~400 µm and ~108 µm. These theoretical values suggest a moderately roughened surface, which may be sufficient to support cell adhesion and tissue integration without inducing excessive inflammation. Still, future studies should incorporate quantitative roughness measurements to correlate topography with biological performance more precisely.

S100B, a calcium-binding protein, plays a key role in cell proliferation, apoptosis, and differentiation both inside and outside cells. Extracellularly, its activity is generally mediated by receptor for advanced glycation end-products (RAGE). S100B has significant trophic effects following damage to the nervous system and skeletal muscles in various cell types, such as astrocytes and microglial cells [36]. After injury, S100B levels increase in the extracellular environment and act as a damage-associated molecular pattern (DAMP). DAMPs are endogenous factors that are visible to the immune system under conditions of cellular stress or damage, thereby initiating sterile inflammation [37].

S100B inhibits the tumor suppressor protein p53, reduces apoptosis, and increases cell proliferation, thereby linking it to certain cancers and their invasiveness. It also inhibits cell differentiation. In the early postnatal period, S100B expression is decreased in astrocytes, followed by re-expression in the final stages of differentiation, suggesting that S100B is crucial for early cell proliferation and migration, but its reduction is necessary for differentiation. S100B has both neurotrophic and neurotoxic effects depending on its concentration, indicating its dual roles [38].

In our study, when the crush nerve injury was first induced, two months after the nerve injury, two months after the application of the PLA tube, and when the biotube was first applied, the S100b value in the serum samples was significantly lower than that in the control. It was thought that the inflammatory response might not have formed because the first blood samples were collected immediately after nerve crush injury. In one study, 24 h post-crush injury, myelinated and unmyelinated fibers in the proximal area appeared normal, while activated Schwann cells involved in myelin degeneration were observed distally. At this stage, S100b protein levels were the same as those in normal nerves [39].

Although the S100b value remained low compared to the control in blood samples taken 2 months after the nerve crush injury and PLA groups, the lack of a significant change in the S100b value compared to the control after biotube treatment suggests that biotubes might have shown a recovery similar to that of an uninjured nerve.

TNF-α (tumor necrosis factor) is a key pro-inflammatory cytokine regulating inflammatory responses after injury and activating other cytokines and growth factors. After peripheral nerve injury, the release of macrophages and Schwann cells triggers a significant inflammatory response [40]. One study found significantly fewer macrophages at the distal injury site after sciatic nerve crush injury in damaged mice [41]. Another study showed that TNF-α inhibits Schwann cell proliferation in a dose-dependent manner without affecting Schwann cell viability [42].

In our study, TNF-α values were significantly higher in all groups than in the control group, except for the group in which the biotube was first applied (group 5). A positive correlation was found between TNF-α and S100b after treatment with PLA tubes. This result indicated that the biotube caused a significant healing process in the injured nerve.

Nerve conduits have long been used, and 3D printers now enhance their production because of their advantages over traditional methods [43,44]. Three-dimensional bioprinting integrates living cells, growth factors, and natural polymers into its design [44]. Key bioprinting components include a tissue/organ 3D model, an aseptic bioprinter, bioink, and cells. A study created a scaffold for nerve regeneration using nano-bioink with Gelatin Methacrylamide, neural stem cells, and graphene nanoplatelets [45].

One study observed significant nerve network formation by adding dopamine to Gelatin Methacrylamide in a 3D-printed scaffold [46]. Another study used 3D-printed conduit tubes made from human dermal fibroblasts in a rat sciatic nerve gap, achieving superior nerve regeneration compared with silicone tubes [44]. Another group used a hydrogel mix of alginate, fibrin, hyaluronic acid, and RGD peptides to produce Schwann cell-covered scaffolds that mimic fibrin clots after nerve injury [27]. Another study created a suitable microenvironment for rat Schwann cells using a composite alginate and gelatin hydrogel [47].

Synthetic nerve conduits pose a risk of infection and have low biocompatibility. Natural polymers are often preferred for the production of nerve guidance conduits (NGCs) because they can be easily degraded by enzymes, exhibit excellent biocompatibility and minimal immunogenicity, and support cell growth [43].

Studies have shown successful axonal growth and nerve regeneration when chitosan was combined with hyaluronic acid [48], used as a gel sponge [27], employed as a nerve conductor [49], compared with muscle-filled veins and autologous nerve grafts [50], and enhanced with bone marrow cells in a 30 mm peroneal nerve defect in goats [51].

Alginate is a biodegradable and biocompatible polysaccharide consisting of mannuronic and glucuronic acids. It can be cross-linked with divalent ions (e.g., Ca^+2^) and used in nerve tissue engineering as a hydrogel or scaffold [52]. A study reported recovery in a 50 mm nerve defect in a cat’s sciatic nerve using alginate [53]. Another study showed motor and sensory nerve reinnervation in a 7 mm nerve defect in rats 18 weeks after surgery, with the regeneration of new myelinated and unmyelinated nerve fascicles. During this time, biodegradable alginate disappeared [54].

The individual contributions of the hydrogel components have been well described in the literature. Chitosan promotes axon elongation and remyelination, and provides mechanical stability and antibacterial activity, although its rigidity and slow degradation may limit flexibility [55]. Alginate has high water content and supports nutrient diffusion, but lacks inherent bioactivity and may degrade unpredictably [56]. Collagen is widely considered the gold standard among natural polymers due to its excellent cell adhesion and bioactivity, although it degrades relatively quickly and may not match the entire timeline of nerve regeneration [57]. The combination of these three materials was intended to synergistically balance these advantages and limitations.

The PLA filament (NatureWorks™ Ingeo 4032D, Minnetonka, MN, USA) used in this study was selected for its biocompatibility, mechanical stability, and well-documented degradation profile. The material has a molecular weight of approximately 200,000 g/mol and a density of 1.24–1.25 g/cm^3^ (NatureWorks LLC, n.d., Minneapolis, MN, USA). Although crystallinity was not directly measured, the printing protocol was optimized to promote a moderate degree of crystallinity (estimated at 35–45%) using a nozzle temperature of 200 °C, a bed temperature of 70 °C, and a closed fan setting during initial layers to reduce cooling rates. The typical crystallinity for 4032D PLA under these conditions is ~35–45%. These settings support moderate crystallization (target ~40%) without excessive brittleness while preserving an amorphous fraction to enhance cell interactions.

The literature indicates that low crystallinity (<20%) may result in premature softening and loss of dimensional stability, while high crystallinity (>50%) can render the structure too rigid and hinder tissue integration by limiting water diffusion and potentially provoking inflammatory responses. The selected PLA formulation provides a favorable balance: it is sufficiently amorphous to permit permeability and support biological interaction, yet crystalline enough to preserve scaffold geometry throughout the regeneration period. This profile aligns well with the expected 2–6 month degradation window for peripheral nerve repair scaffolds [30,58].

A study showed that using multilayered and braided PLA conduits in a 10 mm rat sciatic nerve defect led to nerve tissue formation [59]. Another study reported 82% functional recovery at 18 months in a 20 mm sciatic nerve defect in rabbits using a microporous PLA conduit [60]. A study created a 7 mm defect in the buccal branch of the rat facial nerve, which was repaired using either a PLA tube, a collagen-filled silicone tube, or an autologous nerve graft. Thirteen weeks post-surgery, although the autograft group had the greatest myelin thickness, the PLA group had more myelinated neural fibers in the middle of the regenerated nerve, suggesting that PLA tubes are comparable to autografts in peripheral nerve regeneration [32].

Our results revealed significantly lower TNF-α levels in the biotube group compared to the PLA and untreated injury groups. This finding suggests that the biotube may reduce pro-inflammatory responses, which is consistent with previous reports that natural polymers such as collagen, alginate, and chitosan exhibit immunomodulatory properties and support tissue regeneration [27,32]. In contrast, the PLA group exhibited elevated TNF-α levels, indicating a more pronounced inflammatory reaction. This may be attributed to residual lactic acid degradation products of PLA, known to induce mild inflammatory responses, especially during early phases of implantation [60]. Regarding S100B, a dual-function protein with both neurotrophic and neurotoxic effects depending on concentration, we observed higher levels in the PLA group compared to the biotube group. Elevated S100B levels may reflect ongoing cellular stress or a compensatory trophic response [36,38]. Overall, the combination of reduced TNF-α and normalized S100B levels in the biotube group supports the hypothesis that bioinks composed of ECM-like materials provide a more favorable microenvironment for nerve regeneration compared to synthetic polymers.

One of the key limitations of this study is the absence of a quantitative analysis of the crystallinity of the PLA material used in the 3D-printed conduits. Although printing conditions were selected to favor a moderate crystalline structure, direct measurements such as differential scanning calorimetry (DSC) or X-ray diffraction (XRD) were not performed. Future studies should include these techniques to better correlate the physicochemical characteristics of the material with its in vivo performance.

A limitation of this study is the lack of direct surface roughness measurements for the 3D-printed PLA conduits. Although roughness was theoretically estimated based on printer settings, precise topographical analysis using techniques like AFM or profilometry is needed in future studies to validate these approximations and assess their biological relevance.

Another limitation of this study is the lack of quantitative assessment of the physical properties of the 3D-printed conduits, including surface roughness, porosity, and mechanical strength. Surface topography plays a critical role in modulating cell adhesion, proliferation, and tissue integration, particularly in polymer-based implants. While the PLA conduits used in this study demonstrated acceptable in vivo biocompatibility, further investigation using profilometric or atomic force microscopy (AFM) techniques is warranted. In addition, porosity influences nutrient diffusion and axonal guidance, while mechanical integrity affects surgical handling and structural stability post-implantation. These aspects could be evaluated using scanning electron microscopy (SEM), micro-CT, or tensile testing in future studies. Such analyses would provide valuable insight into the relationship between scaffold architecture and biological response and could guide the optimization of 3D printing parameters for next-generation nerve repair constructs.

Furthermore, this study did not include rheological characterization of the bioink used during bioprinting. Rheological parameters such as shear-thinning behavior, storage modulus (G’), and yield stress have been shown to critically influence print fidelity, cell viability, and post-print structural stability. In the case of hydrogel-based bioinks like alginate and chitosan, the inadequate control of viscosity and elasticity may result in excessive shear stress during extrusion or collapse of the printed construct after deposition. Moreover, the viscoplastic nature of such materials often leads to wall slip effects, requiring advanced characterization methods such as oscillatory rheometry and slip-corrected flow curve analysis. Incorporating these methods in future work will be essential for optimizing both the biological and mechanical performance of printed constructs [61,62,63].

## 5. Conclusions

This study aimed to explore the feasibility of using 3D-printed biocompatible nerve conduits for peripheral nerve regeneration following sciatic nerve injury in a rat model. Our findings demonstrated that bioprinted conduits composed of collagen, alginate, and chitosan provided favorable microenvironments, with reduced TNF-α expression and improved tissue integration compared to PLA-based and untreated groups. These results highlight the potential of customized, hydrogel-based conduits in promoting nerve repair and minimizing inflammation. Overall, these findings serve as a proof-of-principle for the use of 3D-printed PLA-based nerve conduits in peripheral nerve repair, highlighting the potential of additive manufacturing to deliver customizable, biocompatible treatment options.

Looking forward, this approach opens new avenues for designing patient-specific neural scaffolds using bioresponsive materials. Future studies should investigate functional recovery outcomes, mechanical performance, and long-term biocompatibility, as well as optimize printing parameters and bioink formulations using advanced rheological and mechanical assessments. The integration of bioactive cues and controlled degradation profiles may further enhance clinical translation of 3D-bioprinted nerve guidance constructs.

## Figures and Tables

**Table 1:** PLA printing parameters used for conduit fabrication. The presented PLA properties were based on the manufacturer’s technical datasheet (NatureWorks LLC, 4032D grade) and relevant literature values [30].

Parameter	Value
Nozzle diameter	0.40 mm
Nozzle temperature	200 °C
Build plate temperature	60 °C
Print speed	50 mm/s
Layer height	0.20 mm
Infill density	70%
Filament material	Ingeo™ 4032D PLA
Filament density	~1.24–1.25 g/cm^3^
Filament Mw	~200,000 g/mol
Approximate crystallinity	~35–45%

**Table 2:** TNF-α and S100B levels of PLA and biotubes using serum samples and control serum samples for nerve healing.

Group	Group 0	Group 1	Group 2	Group 3	Group 4	Group 5	Group 6	*p*
Variable	*n* = 11	*n* = 5	*n* = 10	*n* = 13	*n* = 8	*n* = 10	*n* = 8	
TNF-α pg/mL	29.91 ± 11.99 a	107.21 ± 41.99 b	220.46 ± 37.54 d	110.82 ± 32.59 b	439.76 ± 80.99 c	17.19 ± 6.88 a	239.07 ± 96.44 d	0.001 **
S100B pg/mL	136.12 ± 43.88 a	29.32 ± 3.96 c	55.11 ± 9.39 b	167.84 ± 36.12 a	58.22 ± 32.18 b	55.11 ± 9.39 b	121.65 ± 16.19 a	0.010 *

Group 0: Control. Group 1: Sera from blood samples collected after nerve damage was created. Group 2: Sera from blood samples collected 2 months after nerve damage was created. Group 3: Sera from blood samples collected after PLA tube insertion. Group 4: Sera from blood samples collected after treatment with the PLA tube. Group 5: Sera from blood samples collected after biotube insertion. Group 6: Sera from blood samples taken after treatment with the biotube. * *p* < 0.05 ** *p* < 0.01. No differences were observed between the same letters. There are differences between different letters.

**Table 3:** Comparison of serum TNF-α and S100B values in PLA and biotube treatment after nerve injury.

TNF-α pg/mL		Group 0	Group 1	Group 2	Group 3	Group 4	Group 5	Group 6
S100B pg/mL	rs	0.330	0.362	−0.094	−0.137	0.777	0.414	0.201
S100B pg/mL	p	0.322	0.549	0.795	0.671	0.023 *	0.234	0.633

Group 0: Control. Group 1: Sera from blood samples collected after nerve damage. Group 2: Sera from blood samples collected two months after nerve damage. Group 3: Sera from blood samples collected after PLA tube insertion. Group 4: Sera from blood samples collected after treatment with the PLA tube. Group 5: Sera from blood samples collected after biotube insertion. Group 6: Sera from blood samples collected after treatment with the biotube. * *p* < 0.05; values are expressed as Spearman’s correlation coefficient (rs) and statistical significance (*p*).

**Table 4:** Intragroup comparison of serum TNF-α and S100B values in PLA and biotube treatment after nerve injury.

TNF-α pg/mL		Group 1 Group 2	Group 3 Group 4	Group 5 Group 6
S100B pg/mL	rs	0.172	−0.033	−0.360
	*p*	0.541	0.889	0.143

Group 1: Sera from blood samples collected after nerve damage. Group 2: Sera from blood samples collected two months after nerve damage. Group 3: Sera from blood samples collected after PLA tube insertion. Group 4: Sera from blood samples collected after treatment with the PLA tube. Group 5: Sera from blood samples taken after biotube insertion. Group 6: Sera from blood samples collected after treatment with the biotube; values are expressed as Spearman’s correlation coefficient (rs) and statistical significance (*p*).

## Data Availability

The data analyzed in this study can be accessed upon request.
